# Time-Aware Graph Neural Network for Asynchronous Multi-Station Integrated Sensing and Communications Fusion in Open RAN

**DOI:** 10.3390/s26082376

**Published:** 2026-04-12

**Authors:** Zhiqiang Shen, Wooseok Shin, Jitae Shin

**Affiliations:** 1 Department of Computer Science and Engineering, Sungkyunkwan University, Suwon 16419, Republic of Korea; zhiqiang001@g.skku.edu; 2 Department of Electrical and Computer Engineering, Sungkyunkwan University, Suwon 16419, Republic of Korea; swsda95@skku.edu

**Keywords:** Integrated Sensing and Communication (ISAC), Open RAN (O-RAN), Age-of-Sensing (AoS), Graph Neural Networks (GNN), asynchronous data fusion, Near-RT RIC, physics-informed learning, network-native sensing, 6G

## Abstract

Multi-station sensing telemetry typically arrives out-of-order at the Open RAN (O-RAN) Near-RT RIC due to non-deterministic jitter in cloud-native protocol stacks, inducing a “temporal scrambling” effect that invalidates traditional spatial fusion. To bridge this gap, we introduce Age-of-Sensing (AoS) as a dynamic reliability metric for asynchronous sensing reports and establish an AoS-aware graph neural network (GNN) paradigm for asynchronous sensing fusion. This paradigm shifts the focus from conventional spatial-only aggregation to time-aware inference by explicitly incorporating sensing freshness into graph-based fusion. As a physics-informed realization of this paradigm, we present Time-Aware Fusion (TA-Fusion), which introduces a TA-Gate mechanism to recalibrate node trust prior to graph aggregation. Unlike passive feature concatenation, the TA-Gate serves as an active gating signal to prioritize fresh telemetry while adaptively suppressing stale outliers. On a standardized O-RAN benchmark, TA-Fusion achieves a root mean square error (RMSE) of 12.22 m, delivering a 21.7% reduction in Mean absolute error (MAE) over the AoS-aware GNN baseline and maintaining robustness in extreme jitter scenarios where traditional linear methods suffer from severe accuracy degradation due to their static weighting logic. Extensive Monte Carlo simulations confirm that the framework preserves consistent error bounds across diverse base station geometries without manual recalibration. These findings support the real-time feasibility of the proposed paradigm for delay-critical Integrated Sensing and Communication (ISAC) services, providing a resilient spatial foundation for 6G orchestration under substantial network-layer jitter.

## 1. Introduction

The transition toward 6G architectures envisions a shift from terminal-centric positioning to *network-native radar sensing*, where the Open RAN (O-RAN) infrastructure treats targets as non-cooperative scattering bodies. Unlike conventional UE-based positioning relying on active Reference Signal Received Power (RSRP) reporting, this paradigm shifts sensing and inference to the network infrastructure, aligning with the 6G vision of a “sensor-free” pervasive sensing environment. We assume that the physical layer (L1) employs orthogonal waveforms or unique sequence identifiers, enabling base stations, acting as O-RAN E2 Nodes, to distinguish reflected signals. This assumption follows standard multi-static radar tracking models and shifts the primary bottleneck from signal identification to the freshness of network-layer sensing information. When non-deterministic network jitter—ranging from 10 to 450 ms—exceeds the sensing reporting interval, the resulting temporal misalignment severely degrades the spatial-temporal coherence required for near-real-time (Near-RT) orchestration in the disaggregated RAN Intelligent Controller (RIC) architecture [1].

To quantify this risk, consider a target moving at 20 m/s. A typical 100 ms jitter induces a 2-m spatial lag—an error exceeding the beamwidth of most mmWave arrays, risking beam misalignment or degraded link tracking. In contrast to centimeter-level radar localization systems designed for autonomous driving, network-native sensing prioritizes infrastructure-level situational awareness across wide coverage areas, where meter-level spatial accuracy is generally sufficient for orchestration and mobility management tasks [2,3]. This sensing paradigm, therefore, complements rather than replaces high-resolution radar localization systems.

Despite the unique advantages of O-RAN sensing, asynchronous data coordination via standardized RIC APIs [4] remains a fundamental challenge in advanced sensing architectures [5], often causing traditional fusion methods to reach a performance bottleneck. For instance, iterative solvers like Geometric Weighted Least Squares (G-WLS) [6] essentially reach a performance floor as they fail to decouple temporal asynchrony from spatial measurement noise, leading to substantial Spatial Mismatch Error (SME). Similarly, state-estimation filters such as the Extended Kalman Filter (EKF) [7] provide temporal smoothing but are susceptible to latency-induced “state-lag” during high-maneuverability segments. At the same time, while Graph Neural Networks (GNNs) [8] are a powerful tool for modeling spatial relationships in multi-node sensing systems, vanilla architectures remain “time-blind”, treating all observations with equal priority regardless of their Age-of-Sensing (AoS). More recently, learning-based approaches have explored improving localization robustness in multistatic sensing environments through signal processing and neural estimation techniques [9]. However, recent studies introducing AoS as an optimization target for resource scheduling in 6G ISAC [10], or applying GNNs to Age-of-Information (AoI)-driven queue management [11], primarily focus on “delay-prevention” through network-layer control. In contrast, the potential of utilizing AoS as a dynamic reliability feature for “delay-resilient” spatial-temporal inference remains largely unexplored, leading to spatial artifacts where stale observations bias the fusion estimate.

To address these challenges, we propose Time-Aware Fusion (TA-Fusion), which introduces a structurally distinct approach to exploiting AoS in graph-based sensing fusion. While prior works either minimize AoS at the network layer or passively append it as an input feature, TA-Fusion treats AoS as an active gating signal that modulates node-level hidden representations after graph attention aggregation. The core mechanism—the Time-Aware Gate (TA-Gate)—combines a physics-motivated exponential decay prior with a learned MLP residual under an explicit monotonicity penalty. This hybrid design ensures that (i) the trust mapping respects the physical principle that older observations carry less spatial reliability, and (ii) the non-linear component can adapt to the complex, non-stationary jitter patterns characteristic of O-RAN E2 backhaul.

The primary contributions of this paper are summarized as follows:

- **Problem Formalization:** We formalize the multi-station sensing fusion challenge under non-deterministic O-RAN jitter by modeling the coupling between AoS and SME. This theoretical bridge reconciles network-layer stochasticity with physical-layer spatial consistency.
- **AoS as an Active Gating Signal:** Unlike prior works that either minimize AoS at the network layer or passively concatenate it as an input feature, we propose treating AoS as a *post-aggregation gating signal* that modulates node-level hidden representations within a GNN. This architectural choice decouples temporal reliability assessment from spatial feature extraction, enabling the graph backbone to focus on geometric reasoning while a dedicated gating module enforces temporal consistency.
- **Hybrid Physics-Informed TA-Gate:** The proposed TA-Gate combines an exponential decay skeleton (capturing the physics of information aging) with a learned MLP residual (adapting to non-stationary jitter patterns), constrained by an explicit monotonicity penalty. This hybrid design is structurally distinct from both pure exponential weighting, which is too rigid for complex jitter dynamics, and unconstrained learned gates, which risk violating the physical monotonicity of information decay. A controlled ablation (Table 5) confirms that neither component alone matches the full hybrid gate.
- **Comprehensive Evaluation:** On a standardized O-RAN benchmark, TA-Fusion reduces MAE by 21.7% over the passive AoS-aware GNN baseline and by 59.9% over the time-blind spatial GNN. Monte Carlo simulations across 100 randomized geometries confirm the lowest error variance (σ=5.08 m) among all methods, demonstrating consistent generalization without per-layout recalibration.
- **Real-time Feasibility:** End-to-end profiling on edge-grade hardware yields an average inference latency of 2.07 ms (P95: 2.35 ms), maintaining a 79.3% timing margin relative to the 10-ms O-RAN Near-RT RIC execution budget.

While the achieved Root mean square error (RMSE) of 12.22 m may not replace control-level maneuvers like emergency braking, it provides crucial safety redundancy and a reliable spatial foundation for application-level tasks such as proactive handover and regional traffic management. Consequently, restoring the network’s independent spatial awareness is a fundamental requirement for the functional stability of 6G cloud-native RAN environments [12].

The remainder of this paper is organized as follows: Section 2 reviews the related work. Section 3 describes the system model and formulates the problem. Section 4 details the proposed TA-Fusion architecture. Section 5 presents the experimental setup, followed by the results and discussion in Section 6. Finally, Section 7 concludes the paper.

## 2. Related Work

In this section, we review the existing literature across three key dimensions: Integrated Sensing and Communication (ISAC) in O-RAN architectures, conventional and learning-based multi-sensor fusion, and the impact of AoS on networked systems.

### 2.1. ISAC and O-RAN Architecture

The ISAC has been identified as a flagship technology for 6G, enabling the dual use of the wireless spectrum for both data transmission and environmental probing [13,14]. The O-RAN Alliance has standardized an open architecture that decouples the control plane from the user plane [1], where the Near-Real-Time (Near-RT) RIC serves as a logically centralized host for ISAC xApps [15]. The practical challenges of the E2 interface, specifically the non-deterministic latency that affects sensing telemetry data, represent an important practical consideration in the current O-RAN ISAC roadmap [16,17]. While localization-assisted beamforming and predictive resource scheduling have been proposed to enhance RAN performance, most studies assume perfect synchronization. Recent studies on cooperative ISAC sensing have also highlighted that infrastructure-based sensing systems primarily target network-scale situational awareness rather than centimeter-level object localization [2,3].

### 2.2. Multi-Sensor Fusion for Localization

Multi-station target tracking has been extensively studied within the framework of classical state estimation [18]. The achievable localization accuracy in cooperative sensing systems is also known to be fundamentally influenced by sensing geometry and synchronization conditions, as analyzed in cooperative ISAC network studies [19]. Classic geometric solvers, particularly G-WLS and its iterative variants, serve as the analytical foundation for multi-sensor aggregation [6]. However, their performance is contingent on a “common time-base” assumption. In the absence of a synchronized clock, these solvers typically reduce to weighted centroids that cannot compensate for the dynamic displacements induced by E2 jitter. While optimal linear filters for Out-of-Sequence Measurements (OOSM) [20] were developed to handle asynchrony via statistical re-alignment, they often require extensive state backtracking or significant computational overhead, which often conflicts with the stringent near-real-time execution requirements of the O-RAN Near-RT RIC.

Recently, GNNs have gained traction for multi-station fusion [21,22], and several studies have explored learning-based approaches to improve localization robustness in multistatic sensing environments [9]. However, existing architectures predominantly assume synchronized sampling or fixed temporal alignment. While some GNN architectures incorporate temporal metadata as a passive input feature—either in graph classification tasks [8] or in adjacent domains such as trajectory prediction [23]—they typically rely on the neural backbone to implicitly learn temporal dynamics without explicit freshness-based trust modulation. In contrast, this study establishes the *AoS-aware GNN paradigm* by implementing an active trust modulation mechanism prior to graph aggregation. By utilizing AoS as an explicit gating signal, the framework ensures that the spatial estimate remains anchored to the true instantaneous geometry, providing a robust benchmark for sensing fusion within the sub-10 ms execution budget required for O-RAN Control Loop 2.

### 2.3. Age of Information (AoI) and Age of Sensing (AoS)

The concept of AoI has been established as a cornerstone metric for quantifying status freshness in networked systems [24,25], spanning from sensing networks to federated learning [26]. Recently, Adhikary et al. [10] formally extended this to the AoS to characterize the informational utility specific to holographic ISAC frameworks.

While network jitter in 5G and Time-Sensitive Networking (TSN) systems is known to cause substantial AoI fluctuations [27], conventional mitigation strategies often rely on online pruning or traffic scheduling at the network layer [28]. In the context of O-RAN, the E2 Application Protocol (E2AP) introduces variable queuing delays that directly inflate the AoS of telemetry reports [4,17]. These existing approaches fall into two categories: (i) *network-layer optimization*, where AoS is treated as a scheduling objective to be minimized [10,11], and (ii) *passive feature inclusion*, where temporal metadata is concatenated as an additional input dimension to learning-based estimators—as practiced in spatial graph networks [21] and trajectory prediction architectures [23]—without structural guarantees on how it shapes the output. Neither category provides an *active, architecturally enforced* mechanism that translates AoS into a trust modulation signal with built-in physical constraints. TA-Fusion addresses this gap by introducing AoS as a post-aggregation gating signal within a GNN architecture, combining a physics-motivated exponential prior with a learned non-linear residual under an explicit monotonicity constraint.

### 2.4. Localization-Assisted Network Optimization

Beyond tracking, UE localization plays a pivotal role in optimizing RAN performance. Recent studies [29,30] have investigated multi-tier multi-user networks where Rate-Splitting Multiple Access (RSMA) and Deep Reinforcement Learning (DRL) are employed to enhance spectral efficiency. However, such advanced optimization frameworks typically assume perfect spatial-temporal synchronization. In cloud-native O-RAN deployments, the non-deterministic asynchrony between E2 reporting and RIC processing remains a critical bottleneck that invalidates traditional “instantaneous state” assumptions. TA-Fusion addresses these challenges by facilitating temporally aligned localization through its AoS-aware architecture. By mitigating the “ghosting” effects of stale telemetry, the framework provides a resilient spatial foundation for downstream 6G tasks, including predictive beam-tracking, proactive handover orchestration, and cooperative perception in stochastic, cloud-native environments.

## 3. System Model and Problem Statement

### 3.1. O-RAN-Based Multi-Station ISAC Architecture

**Figure 1:**
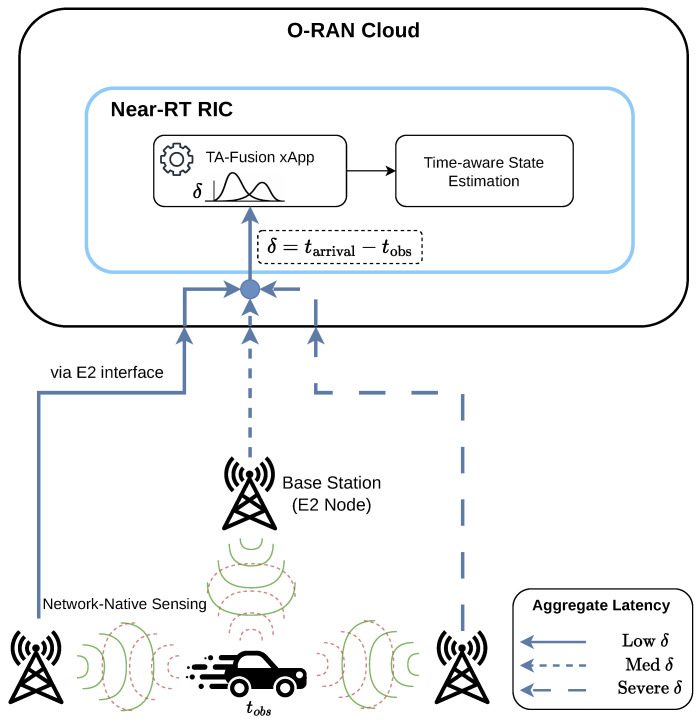
O-RAN Near-RT RIC architecture for network-native ISAC. The xApp mitigates heterogeneous E2 interface latency ($\delta =t_{\mathrm{arrival}}-t_{\mathrm{obs}}$) across BS-RIC links with varying congestion levels (Low, Med, Severe δ) to ensure time-aware state estimation.

Within the proposed *AoS-aware GNN architectural paradigm*, we consider an O-RAN-native multi-station sensing network deployed for target localization and tracking, as illustrated in Figure 1. The system consists of a set of *N* Base Stations (BSs), denoted as $B=\{b_{1},b_{2},\ldots ,b_{N}\}$, each located at a fixed coordinate $a_{i}={\left[x_{BS,i},y_{BS,i}\right]}^{T}$ within a sensing region $A\subset R^{2}$. To bridge the gap between network-layer stochasticity and physical-layer consistency, the Near-Real-Time (Near-RT) RIC hosts the TA-Fusion framework as an xApp, which aggregates asynchronous telemetry data via the E2 interface.

Crucially, this architecture treats the AoS not merely as a timestamp, but as a *fundamental reliability metric*. During the offline training phase, ground-truth (GT) target positions p(t) are obtained via high-precision reference systems to supervise the learning of the TA-Gate’s decay logic. In the online inference phase, the paradigm relies solely on the asynchronous telemetry $z_{i}$ and its corresponding AoS metadata $\tau_{i}$ arriving at the Near-RT RIC. As illustrated in Figure 1, the communication links between the E2 Nodes and the Near-RT RIC are subject to heterogeneous backhaul latency $\delta_{i}$, ranging from low to severe congestion levels. The proposed paradigm is specifically designed to reconcile these temporally scrambled telemetry streams, restoring a coherent spatial state from mutually incoherent temporal observations.

### 3.2. Waveform and Data Association Assumptions

To focus on the higher-layer intelligent fusion logic, we assume that the underlying physical layer (L1) utilizes orthogonal waveforms or unique sequence IDs (e.g., distinct Zadoff-Chu sequences). This orthogonality ensures that each Base Station (E2 Node) $b_{i}$ can uniquely identify the reflected echo signals originating from the target.

This assumption is standard in multi-static radar and ISAC tracking literature [14,22], where the research focus shifts from low-level signal detection to high-layer spatial-temporal fusion. Consequently, the data association challenge—mapping specific measurements to their respective source nodes—is assumed to be resolved at the E2 Node level through established filtering techniques. This assumption allows us to abstract away physical-layer impairments such as clutter, false alarms, or missed detections, which are orthogonal to the network-layer asynchrony problem addressed in this work. Specifically, we assume that each E2 Node $b_{i}$ is equipped with basic L1–L2 processing capabilities (e.g., CFAR detection and centroid estimation) to generate a local coordinate estimate $z_{i}$ prior to transmission. By treating the E2 Node as a “reliable local estimator,” the research focus is narrowed to the higher-layer fusion bottleneck. We emphasize that this coordinate-level abstraction is a deliberate methodological choice to decouple physical-layer detection uncertainty from network-layer asynchrony, ensuring that the observed precision gains are attributable to the resolution of temporal asynchrony rather than varying L1 sensing capabilities. We acknowledge that the orthogonal waveform assumption may face limitations in ultra-dense or interference-limited deployments. However, recent O-RAN ISAC studies [2,14] have demonstrated that waveform orthogonality is practically achievable in typical macro-cell configurations (N≤16) using current 5G NR signaling, and the proposed framework can accommodate imperfect data association by treating it as an additional noise source in the measurement model of Equation (4).

### 3.3. Target Mobility and Measurement Model

To evaluate the system under high-dynamic conditions and assess generalization, we model the target trajectory $p\left(t\right)={\left[x\left(t\right),y\left(t\right)\right]}^{T}$ using three distinct mobility patterns:

1. **Eight-figure (lemniscate):** A complex periodic pattern characterized by frequent maneuvers and varying velocities, used as the primary training scenario:
  (1) $$\left\{\begin{array}{c} x\left(t\right)=x_{center}+A_{x}\sin\left(\omega t\right)\\ y\left(t\right)=y_{center}+A_{y}\sin\left(2\omega t\right) \end{array}\right.$$
  where $\left[x_{center},y_{center}\right]$ is the area center, $A_{x},A_{y}$ are the amplitudes, and ω is the angular frequency.
2. **Zig-zag (piecewise linear):** A transitional pattern representing sudden directional changes at fixed intervals ΔT:
  (2) $$p\left(t\right)=p\left(t-\Delta t\right)+v_{\theta }\Delta t$$
  where the velocity vector $v_{\theta }$ undergoes a sharp heading rotation $\theta \in [\pm 45^{\circ },\pm 90^{\circ }]$ every 2 s.
3. **Random walk (stochastic):** A non-deterministic pattern representing unpredictable motion:
  (3) $$p_{k+1}=p_{k}+u_{k},\;u_{k}∼N\left(0,Q\right)$$
  where $u_{k}$ is the displacement noise with covariance Q.

At each observation timestamp $t_{obs}$, BS $b_{i}$ performs a local sensing estimation $z_{i}\left(t_{obs}\right)$. We define $z_{i}$ as coordinate telemetry, which represents the target’s position in the global spatial frame as perceived by the corresponding graph node $v_{i}$ (where each BS $b_{i}$ maps to a node $v_{i}\in V$) via local geometric inversion (e.g., trilateration or DOA estimation). This allows the Near-RT RIC to treat each incoming report as a spatially-tethered likelihood constraint originating from anchor $a_{i}$. By linking the reported coordinate $z_{i}$ to its physical anchor, we define a geometric manifold where fusion is treated as a spatial consensus problem. Importantly, this formulation accounts for heterogeneous spatial uncertainty, where the reliability of $z_{i}$ is coupled to the target-anchor geometry, providing a consistent framework for both neural aggregation and non-linear solvers. The measurement model is defined as follows:

(4) $$z_{i}\left(t_{obs}\right)=p\left(t_{obs}\right)+n_{i}$$

where $n_{i}∼N\left(0,\sigma^{2}I_{2}\right)$ represents the additive white Gaussian noise (AWGN). This measurement model is a standard assumption in the literature for point-target tracking in cluttered environments [18]. As specified in Table 1, we maintain a consistent noise level across all nodes to isolate the impact of network-layer latency and temporal asynchrony.

To bridge the continuous target dynamics with the discrete-time fusion process at the Near-RT RIC, we let $t_{k}$ denote the *k*-th fusion instant. The ultimate goal of TA-Fusion is to estimate the instantaneous target state $p(t_{k})$ (hereafter abbreviated as $p_{k}$) by aggregating telemetry reports collected up to the arrival time $t_{arrival}\leq t_{k}$.

### 3.4. Network Jitter and Age-of-Sensing (AoS)

The fundamental challenge addressed here is not conventional measurement noise, but systemic *temporal aliasing* occurring within the cloud-native O-RAN protocol stack. In a practical O-RAN deployment, sensing reports are typically delivered via the E2 report service model. To support high-mobility tracking, each Base Station (E2 Node) $b_{i}$ performs sensing measurements at a rapid periodic interval $T_{s}=20$ ms (i.e., $f_{s}=50$ Hz). However, these reports are not delivered to the Near-RT RIC instantaneously. A sensing report generated at the E2 Node at time $t_{obs}$ traverses the O-RAN backhaul with variable delay, arriving at the Near-RT RIC at time $t_{arrival}=t_{obs}+\delta_{proc}+\delta_{jitter}$. Here, $\delta_{proc}$ represents the deterministic protocol overhead, while $\delta_{jitter}$ is the non-deterministic queuing delay.

For a fusion task triggered at the RIC at any snapshot *k* (corresponding to time $t_{k}$), a report is available for processing if and only if $t_{k}\geq t_{arrival}$. This leads to the fundamental paradox of O-RAN sensing: the “freshest” available report in the RIC buffer is not necessarily the one with the largest $t_{obs}$, but the one that suffered the least backhaul jitter. The causal lifecycle of a sensing report is thus governed by the aggregate latency $\delta =t_{\mathrm{arrival}}-t_{\mathrm{obs}}$, which decomposes into $\delta =\delta_{\mathrm{proc}}+\delta_{\mathrm{jitter}}$. While $\delta_{proc}$ is typically deterministic, the stochasticity of $\delta_{jitter}$ creates a non-linear “waiting time” for the RIC fusion task. By utilizing AoS as an ex-ante metric, our framework remains agnostic to the specific internal distribution of these delays, focusing instead on their combined impact on spatial-temporal consistency.

A critical architectural challenge arises when the aggregate latency significantly exceeds the reporting interval, i.e., $\delta \gg T_{s}$. In congested O-RAN scenarios, while the nominal latency often stays within 150 ms, extreme backhaul bursts can push δ up to 450 ms, equivalent to a lag of over 22 reporting cycles. This leads to a severe “temporal aliasing” problem at the Near-RT RIC: multiple reports from the same BS may arrive out-of-order, or the most recent available data may be significantly stale, causing spatially inconsistent state estimates at the fusion center.

For a fusion task initiated at the Near-RT RIC at snapshot *k*, the freshness of the most recent observation from BS $b_{i}$ is quantified by the physical AoS $\tau_{i,k}=t_{k}-t_{obs}^{\left(i\right)}$, representing the physical time elapsed since measurement, defined as follows:

(5) $$\tau_{i,k}=t_{k}-t_{obs}^{\left(i\right)},\;s.t.\;t_{k}\geq t_{arrival}^{\left(i\right)}$$

where $\tau_{i,k}$ is the discrete realization of the continuous AoS function $\tau_{i}\left(t\right)=t-t_{obs}^{\left(i\right)}$ evaluated at the fusion instant $t=t_{k}$.

In this study, AoS is utilized as a proxy for measurement age, primarily capturing the aggregate latency δ whose stochasticity is dominated by non-deterministic E2 interface jitter $\delta_{jitter}$. To focus on the impact of network-layer asynchrony, we assume that sensing processing at the E2 Node and fusion computation at the Near-RT RIC are near-instantaneous relative to the backhaul jitter, and thus are not explicitly modeled in the AoS calculation. However, in scenarios where physical-layer sensing processing (e.g., radar range–Doppler Fast Fourier Transform (FFT) processing and Constant False Alarm Rate (CFAR) target detection) is non-negligible, such deterministic delays can be readily incorporated into the cumulative AoS calculation or treated as an independent metadata channel within the TA-Gate mechanism to maintain spatial-temporal alignment.

**Figure 2:**
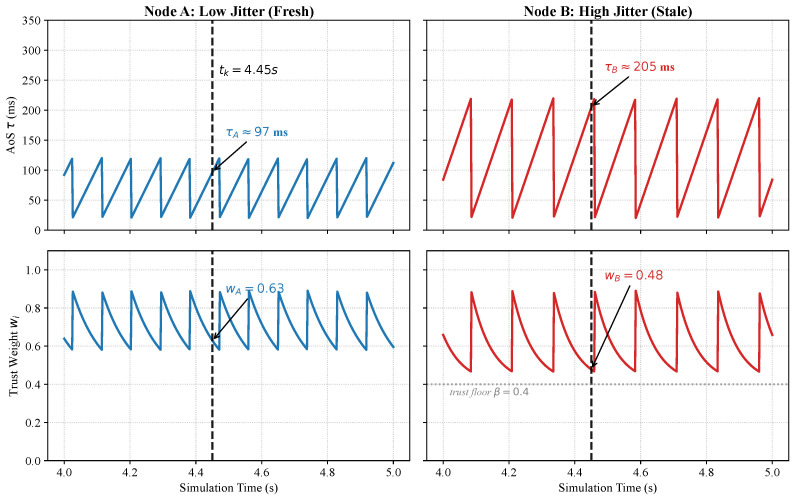
Temporal dynamics of AoS and corresponding trust recalibration. (**Top**) Stochastic E2 latency induces asymmetric AoS fluctuations ($\tau_{A}\approx 97$ ms vs. $\tau_{B}\approx 205$ ms) at the fusion snapshot $t_{k}$. (**Bottom**) The TA-Gate recalibrates node influence based on informational freshness, where the trust floor β=0.4 mitigates complete informational loss for stale observations ($w_{A}=0.63,$ $w_{B}=0.48$). Trust curves illustrate the monotonic decay prior incorporated within the physics-informed architecture.

As illustrated in Figure 2, the AoS profile follows a periodic sawtooth pattern, where the peak amplitude represents the maximum informational staleness experienced by each node. To visualize the gating logic in a real-time context, consider the fusion instant $t_{k}=4.45$ s (indicated by the vertical dashed line). At this specific timestamp, the two nodes exhibit distinct reliability profiles: Node A maintains a relatively fresh state ($\tau_{A}\approx 97$ ms), resulting in a higher trust weight ($w_{A}=0.63$). In contrast, Node B suffers from a significant backhaul burst, with its AoS peaking at $\tau_{B}\approx 205$ ms, which triggers the TA-Gate to suppress its influence to $w_{B}=0.48$. This disparity illustrates the framework’s capacity for instantaneous quality-of-information (QoI) discrimination. By adaptively attenuating nodes with high SME, like Node B, TA-Fusion is intended to prevent stale outliers from inducing trajectory warping, thereby preserving the spatial integrity of the global fusion result.

*Unit and scaling convention:* Throughout this paper, τ (measured in seconds) denotes the physical Age-of-Sensing. To facilitate numerical stability and gradient flow, the neural network operates on the normalized AoS, defined as ${\tilde{\tau }}_{i,k}=\gamma \tau_{i,k}$, where γ=10.0 is the normalization factor. All gating functions w(·) and network inputs are formulated with respect to the dimensionless feature $\tilde{\tau }$ (e.g., $\tilde{\tau }\in \left[0,4.5\right]$ for a maximum aggregate latency of 450 ms), while millisecond (ms) notation is reserved for descriptive contexts and axis labels.

*Promoting spatial consistency:* In this framework, we promote *spatial consistency* by adopting a monotonic trust prior—where the fusion weight assigned to a sensing report is a non-increasing function of its AoS. This monotone law serves as a sufficient inductive bias to facilitate a global estimate that remains anchored to fresh telemetry, thereby mitigating the “ghosting” artifacts and trajectory warping that typically occur when a model over-relies on stale observations.

### 3.5. Problem Statement

The objective of this study is to design a fusion mapping function Φ(·) that estimates the real-time position ${\hat{p}}_{k}$ of the target at the fusion snapshot *k* (corresponding to time $t_{k}$). In this context, we formalize *spatial consistency* as the capacity to reconstruct a temporally coherent state ${\hat{p}}_{k}$ from observations that are mutually incoherent in the temporal domain (i.e., $\left\{t_{obs,i}\right\}$ are non-identical and out-of-order).

Operationally, Φ achieves spatial consistency if it mitigates the trajectory warping caused by stale telemetry—promoting an estimate ${\hat{p}}_{k}$ that represents the target’s physical location at the snapshot *k*, rather than a “ghost” artifact biased by historical, latency-affected reports. Let $Z_{k}=\left\{z_{1},\ldots ,z_{N}\right\}$ be the most recent measurements available at snapshot *k* and $T_{k}$ be their corresponding AoS values. The optimization problem is formulated as follows:

(6) $$\min_{\Phi }E\left[∥p_{k}-\Phi \left(Z_{k},T_{k},G\right){∥}^{2}\right]$$

subject to the graph topology G and the monotonicity of information decay. We formalize the SME as the displacement between a stale observation and the target’s true instantaneous state. By minimizing the Mean Squared Error (MSE) in (6), TA-Fusion seeks to mitigate the aggregate SME, thereby reducing the non-physical “dragging effect” (trajectory warping) in the estimated path. Consequently, the reported RMSE in Section 6.1 serves as a directional operational surrogate for the framework’s capacity to suppress SME. While RMSE is a generic precision metric, its reduction in this context measures the residual spatial displacement induced by temporal aliasing. Therefore, significant gain during high-dynamic transients—where temporal incoherence physically manifests as path warping—serves as empirical support for the framework’s capacity to restore spatial-temporal coherence through time-aware gating.

## 4. The TA-Fusion Architecture

### 4.1. Framework Overview

Building upon the *AoS-aware GNN paradigm* established in Section 3.5, TA-Fusion is designed as a physics-informed, gating-augmented framework for asynchronous telemetry fusion. The core innovation lies in the TA-Gate module, which moves beyond conventional passive feature concatenation by implementing an active trust modulation mechanism. This module computes a per-node trust weight from the AoS metadata, then applies it to the learned hidden representations after graph attention aggregation but before global pooling, ensuring that each node’s contribution to the final estimate is re-weighted by its temporal reliability.

**Figure 3:**
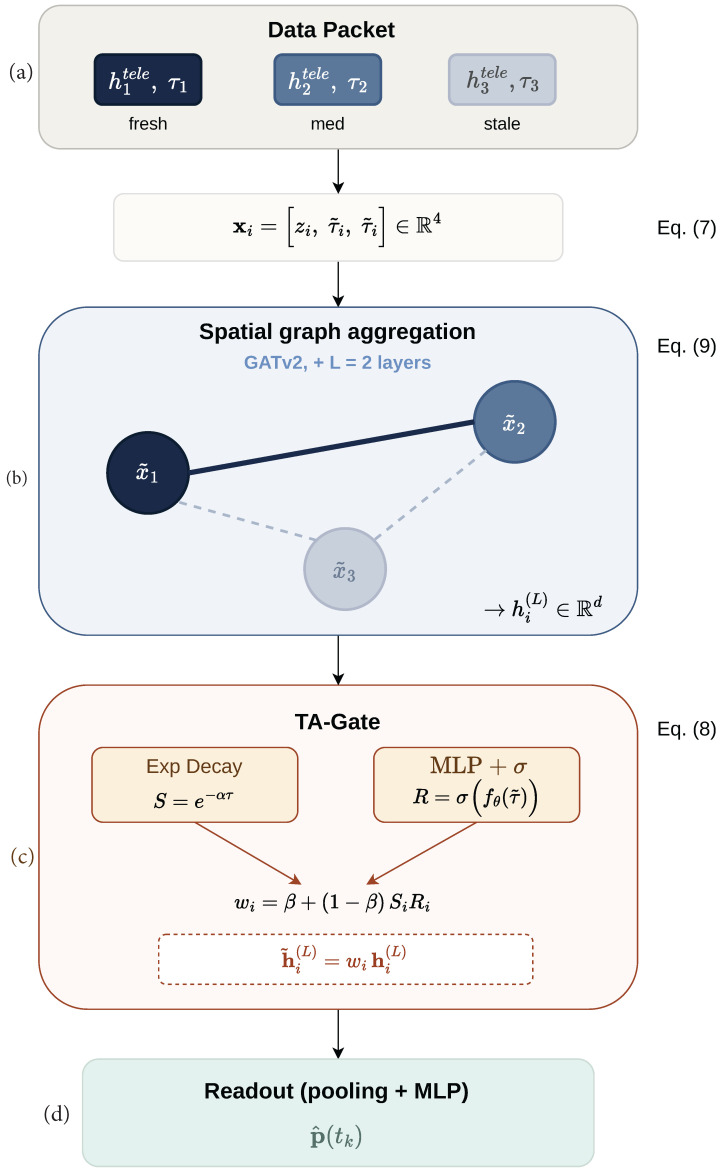
Architectural schematic of TA-Fusion: (**a**) ingestion of raw telemetry $\left[z_{i},{\tilde{\tau }}_{i}\right]$; (**b**) graph attention aggregation (L=2 GATv2 layers) over *k*-NN topology to produce node-level hidden representations; (**c**) post-aggregation TA-Gate modulation, where physics-informed trust weights $w_{i}$ re-scale each node’s hidden state based on AoS; (**d**) global mean pooling followed by MLP readout for target coordinate regression $\hat{p}\left(t_{k}\right)$.

As illustrated in the architectural schematic (Figure 3), the workflow realizes the time-aware inference logic through three primary stages: (1) graph construction, where raw, temporally scrambled telemetry is mapped onto an undirected *k*-NN graph G; (2) spatial aggregation, where multi-layer graph attention networks extract node-level spatial representations; and (3) TA-Gate modulation, where the learned hidden states are re-weighted by physics-informed trust scores prior to global pooling and coordinate regression. The real-time execution logic, which ensures deterministic performance within O-RAN timing constraints, is detailed in Algorithm 1.
**Algorithm 1** TA-Fusion Inference for Asynchronous O-RAN Sensing**Require:** Raw telemetry ${\left\{\left(z_{i,k},\tau_{i,k}\right)\right\}}_{i=1}^{N}$, BS anchors ${\left\{a_{i}\right\}}_{i=1}^{N}$, safety margin β, gating parameters (α,θ) **Ensure:** Synchronized target position estimate ${\hat{p}}_{k}$
  1:**Step 1: Graph Construction**  2:Construct *k*-NN adjacency set E using Euclidean distances $\mathrm{dist}(a_{i},a_{j})$.  3:**Step 2: Feature Construction**  4:**for** i←1 **to** *N* **do**  5:   ${\tilde{\tau }}_{i,k}\leftarrow \gamma \cdot \tau_{i,k}$                      ▹ Normalized AoS feature  6:   $x_{i,k}\leftarrow {\left[z_{i,k}^{⊤},\;{\tilde{\tau }}_{i,k},\;{\tilde{\tau }}_{i,k}\right]}^{⊤}\in R^{4}$         ▹ Node feature with duplicated AoS  7:**end for**  8:**Step 3: Graph Attention Aggregation**  9:$H_{k}^{\left(L\right)}\leftarrow \mathrm{GATv}2\left(X_{k},E;W\right)$       ▹*L* layers of attention-based message passing10:**Step 4: TA-Gate Trust Modulation**11:**for** i←1 **to** *N* **do**12:   $w_{i,k}\leftarrow \beta +\left(1-\beta \right)\cdot \left[\exp\left(-\alpha \tau_{i,k}\right)\cdot \sigma_{\mathrm{sig}}\left({\mathrm{MLP}}_{gate}\left({\tilde{\tau }}_{i,k};\theta \right)\right)\right]$13:   ${\tilde{h}}_{i,k}^{\left(L\right)}\leftarrow w_{i,k}\cdot h_{i,k}^{\left(L\right)}$              ▹ Post-aggregation trust re-weighting14:**end for**15:**Step 5: Readout**16:$h_{final,k}\leftarrow \mathrm{GlobalMeanPooling}\left({\tilde{H}}_{k}^{\left(L\right)}\right)$17:${\hat{p}}_{k}\leftarrow \mathrm{MLP}\left(h_{final,k}\right)$             ▹ Coordinate regression **return** ${\hat{p}}_{k}$


### 4.2. Graph Representation of Asynchronous Sensing

At any fusion snapshot *k* (corresponding to time $t_{k}$), the sensing network is modeled as an undirected graph $G_{k}=\left(V,E\right)$ constructed via symmetric *k*-NN connectivity based on inter-anchor distances. The node feature vector fed into the GNN backbone is defined as follows:

(7) $$x_{i,k}={\left[\underset{\mathrm{Position}\;\left(2-D\right)}{\underset{︸}{z_{i,k}^{T}}},\underset{\mathrm{AoS}\;\left(1-D\right)}{\underset{︸}{{\tilde{\tau }}_{i,k}}},\underset{\mathrm{Gate}\;\mathrm{Input}\;\left(1-D\right)}{\underset{︸}{{\tilde{\tau }}_{i,k}}}\right]}^{T}\in R^{4}$$

where ${\tilde{\tau }}_{i,k}=\gamma \tau_{i,k}$ is the normalized AoS with γ=10.0. The AoS is intentionally duplicated: the third dimension serves as a learnable temporal feature for the GNN attention mechanism, while the fourth dimension is reserved as a dedicated input channel for the TA-Gate’s trust computation (Section 4.3). This duplication ensures that the graph attention layers and the gating module can develop independent representations of temporal freshness without competing for the same feature channel. The factor γ scales the physical AoS into a numerically active range (${\tilde{\tau }}_{i,k}\in \left[0,4.5\right]$), preventing gradient saturation while preserving sensitivity to fine-grained temporal variations across the 10–450 ms latency spectrum.

Critically, the TA-Gate operates as a *post-aggregation* module: the raw features $x_{i,k}$ are first processed by the GNN backbone to produce spatially enriched hidden representations $h_{i,k}^{\left(L\right)}$, which are then re-weighted by the trust scores $w_{i,k}$ computed from the AoS channel. This design allows the graph attention layers to perform unbiased spatial reasoning over all nodes, while the TA-Gate subsequently suppresses contributions from nodes whose temporal staleness implies high spatial mismatch error.

### 4.3. TA-Gate Mechanism

The core innovation of this work is the TA-Gate mechanism, designed to mitigate “temporal aliasing” when aggregate latency $\delta >T_{s}$. As established in Section 3.4, in such regimes, the RIC receives telemetry that is no longer spatially coherent with the target’s current position. In the TA-Fusion architecture (Figure 3), the TA-Gate acts as a learnable post-aggregation filter that re-weights each node’s hidden representation according to its temporal reliability.

For each node $v_{i}$ at snapshot *k*, the TA-Gate computes a trust weight $w_{i,k}\in \left[\beta ,1\right]$ using a hybrid physics-learned architecture:

(8) $$w_{i,k}=\beta +\left(1-\beta \right)\cdot \underset{g(\tau_{i,k})}{\underset{︸}{\left[\exp\left(-\alpha \tau_{i,k}\right)\cdot \mathrm{Sigmoid}\left({\mathrm{MLP}}_{gate}\left({\tilde{\tau }}_{i,k}\right)\right)\right]}}$$

where β=0.4 is the safety margin, $\tau_{i,k}$ is the physical AoS, and ${\tilde{\tau }}_{i,k}=\gamma \tau_{i,k}$ is the normalized AoS input.

This design promotes both physical interpretability and representative flexibility. The exponential term $\exp(-\alpha \tau_{i,k})$ serves as a *deterministic physics-skeleton*, capturing the macro-level informational decay inherent in moving-target sensing. Simultaneously, the ${\mathrm{MLP}}_{gate}$ component acts as a learnable calibration residual, adaptively fine-tuning the trust weight to account for non-linearities induced by the O-RAN protocol stack and containerized processing fluctuations. By operating on the normalized feature ${\tilde{\tau }}_{i,k}$, the gating mechanism is structured to remain responsive to subtle AoS variations across the entire 10–450 ms latency spectrum while upholding numerical consistency.

By re-weighting $h_{i,k}^{\left(L\right)}$ with $w_{i,k}$ after spatial aggregation, the framework selectively suppresses E2 Nodes suffering from extreme latency while the graph topology—constructed from the fixed anchors $a_{i}$—preserves the spatial structure. This mitigates the trajectory bias caused by stale observations pulling the estimate toward outdated positions, promoting a global fusion state dominated by the most reliable reports.

### 4.4. Neural Backbone and Spatial Aggregation

The computational backbone of TA-Fusion employs L=2 layers of Graph Attention Networks (GATv2) [31]. We select GATv2 over standard GCN because its dynamic attention mechanism can implicitly learn to weight message-passing contributions from neighboring nodes, complementing the explicit temporal gating provided by the TA-Gate. For a given fusion snapshot *k*, the hidden state update for node $v_{i}$ at layer l+1 is:

(9) $$h_{i,k}^{\left(l+1\right)}=\sigma \left(\sum_{j\in N(i)\cup \{i\}}\alpha_{ij}^{\left(l\right)}W^{\left(l\right)}h_{j,k}^{\left(l\right)}\right)$$

where $\alpha_{ij}^{\left(l\right)}$ are learned attention coefficients computed via the GATv2 scoring function $\alpha_{ij}^{\left(l\right)}={\mathrm{softmax}}_{j}\left(a^{T}\mathrm{LeakyReLU}\left(W^{\left(l\right)}\left[h_{i,k}^{\left(l\right)}∥h_{j,k}^{\left(l\right)}\right]\right)\right)$, and $h_{j,k}^{\left(0\right)}=x_{j,k}$ is the input feature vector. The first layer uses 4 attention heads (concatenated), while the final layer uses a single head.

After *L* layers, the TA-Gate modulates the resulting hidden representations by the trust weight: ${\tilde{h}}_{i,k}^{\left(L\right)}=w_{i,k}\cdot h_{i,k}^{\left(L\right)}$, where $w_{i,k}$ is computed from Equation (8). This post-aggregation gating ensures that each node’s spatially enriched representation is rescaled according to its temporal reliability before contributing to the global estimate. The modulated states are then aggregated via global mean pooling to form $h_{final,k}$, which is projected by a two-layer MLP (128 → 64 → 2) to regress the target coordinates ${\hat{p}}_{k}$.

*Computational Complexity:* Following the standard formulation for Message Passing Neural Networks (MPNNs), the total inference complexity per snapshot is:

(10) $$O\left(L\cdot (|E|d+Nd^{2})\right)$$

where *N* is the number of base stations, |E| is the number of edges, *d* is the hidden dimension, and *L* is the number of layers. In our O-RAN configuration, the edge set is constructed via *k*-NN such that |E|≈Nk. Given the fixed parameters of N=8, k=4, d=128, L=2, the graph operations remain within a constant and small-scale regime. This linear scaling facilitates millisecond-level execution while remaining theoretically scalable for higher-density 6G sensing scenarios.

### 4.5. Physics-Constrained Optimization and Monotonicity Penalty

To promote a TA-Gate that adheres to the physical principle of information decay, we introduce a *Monotonicity Penalty*. This term serves as the structural enforcer of spatial consistency, formulated within the total loss $L_{total}$ for a given batch of *M* snapshots as follows:

(11) $$L_{total}=\underset{L_{MSE}}{\underset{︸}{\frac{1}{M}\sum_{k=1}^{M}{∥p_{k}-{\hat{p}}_{k}∥}^{2}}}+\lambda \underset{L_{mono}}{\underset{︸}{\sum_{j=1}^{K-1}\mathrm{ReLU}\left(w\left({\hat{\tau }}_{j+1}\cdot \gamma \right)-w\left({\hat{\tau }}_{j}\cdot \gamma \right)\right)}}$$

where $L_{mono}$ quantifies the violation of physical consistency across a predefined set of K=4 sampling points $\left\{{\hat{\tau }}_{j}\right\}=\left\{0,0.1,0.2,0.45\right\}$ s covering the operational AoS support [0,450] ms. To ensure cross-phase consistency, we apply the identical normalization factor γ=10.0 to both the penalty sampling and real-time inference.

*Justification of the monotonicity prior:* The constraint that w(τ) is non-increasing in τ encodes a minimal physical assumption: a sensing report cannot become *more* spatially reliable as it ages, because the target’s displacement from the observed position can only grow (or stay constant) with elapsed time. This assumption holds for any target with bounded velocity, and does not require knowledge of the specific motion model. While we do not claim formal optimality guarantees, the monotonicity penalty acts as a soft inductive bias—it restricts the hypothesis space of the learned gate to physically plausible mappings without imposing a rigid functional form. The gate component ablation in Table 5 empirically validates this design: the MLP-only gate (without monotonicity) achieves comparable accuracy but higher trajectory roughness, confirming that the penalty provides structural regularization beyond what the data-driven component alone can learn.

**Figure 4:**
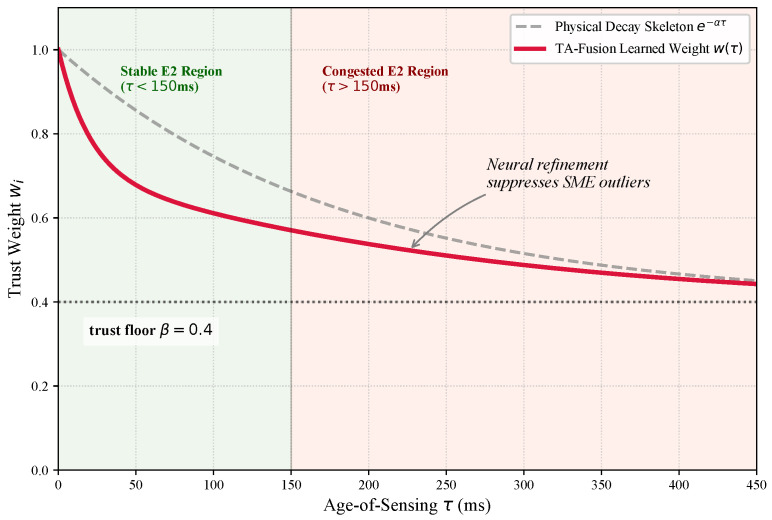
Learned trust decay w(τ) of the TA-Gate. The hybrid mechanism (red line) adaptively refines the exponential skeleton (dashed line) to proactively suppress stale telemetry. A trust floor β=0.4 maintains BS anchors as essential geometric safeguards during congestion (τ>150 ms), mitigating trajectory warping (SME) through accelerated neural attenuation.

This penalty forces the network to converge toward a strictly non-increasing trust decay curve, preventing overfitting to transient jitter patterns. As illustrated in Figure 4, the learned gating profile (red solid line) demonstrates a distinct departure from the physical skeleton even within the stable regime. This early divergence indicates that the TA-Gate identifies the risks of SME as more severe than predicted by a standard exponential model, especially in high-mobility scenarios. Upon entering the congested regime (τ>150 ms), the trust weight asymptotically approaches the trust floor β. This “soft-landing” behavior confirms the framework’s capacity to aggressively marginalize SME-heavy outliers while preserving the structural integrity of the graph topology for localization. By explicitly incorporating this physics-informed prior, TA-Fusion captures a transferable decay prior that remains highly interpretable across heterogeneous network conditions.

## 5. Experimental Setup

### 5.1. O-RAN Simulation Environment

To evaluate the performance and generalization capability of the proposed TA-Fusion framework, we developed a high-fidelity O-RAN sensing simulator. The simulation environment models a 1000×1000 m square area where N=8 Base Stations (BSs) are deployed as E2 Nodes.

To rigorously test the framework’s geometry-specific generalization, we distinguish between a fixed “Benchmark Layout” and “Randomized Topologies.” While the model is trained under a diverse set of jitter conditions, its spatial awareness is evaluated across 100 completely unseen BS configurations. Crucially, the testing phase maintains the same underlying bimodal jitter distribution and sensing graph size (N=8) as the training phase, focusing on the model’s ability to generalize across randomized spatial coordinates without retraining.

**Figure 5:**
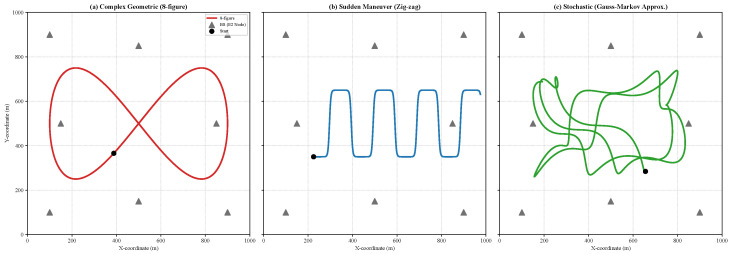
Visualization of the O-RAN simulation environment and the evaluated mobility models: (**a**) 8-figure trajectory; (**b**) zig-zag trajectory; (**c**) stochastic random walk.

As illustrated in Figure 5, we evaluate the framework across three distinct mobility patterns: (a) a complex “8-figure” (lemniscate) trajectory, (b) a sudden zig-zag maneuver, and (c) a stochastic random walk (Gauss-Markov process). In all scenarios, the target maintains a velocity of 15 to 25 m/s, representing a high-mobility context where the combination of high-frequency sampling and bimodal network jitter makes temporal asynchrony most pronounced.

The backhaul network is simulated with non-deterministic Aggregate E2 Latency. Following the heterogeneous network conditions in [17,32], we model the latency δ as a bimodal Gamma mixture: $p\left(\delta \right)=\pi \Gamma \left(k_{1},\theta_{1}\right)+\left(1-\pi \right)\Gamma \left(k_{2},\theta_{2}\right)$, where π=0.6. The first component represents low-latency conditions (shape $k_{1}=2$, scale $\theta_{1}=15$), while the second models congestion bursts ($k_{2}=15,\theta_{2}=20$). To reflect protocol-level timeouts and minimum stack overhead, the distribution is truncated to the range [10,450] ms. While this i.i.d. model serves as a rigorous baseline for testing out-of-order resilience, it provides the necessary stochastic stress to evaluate the TA-Gate’s response to extreme temporal aliasing.

The sensing telemetry is reported by the E2 Nodes via the E2 report service with a unified periodic interval of $T_{s}=20$ ms, which is consistent with the O-RAN Loop 2 control requirements for high-mobility target tracking. Under this configuration, the non-deterministic backhaul latency δ (ranging up to 450 ms) frequently exceeds the reporting interval ($\delta \gg T_{s}$), thereby creating the severe asynchrony and out-of-order delivery that TA-Fusion is designed to mitigate.

### 5.2. Parameter Settings

The key simulation and hyper-parameters used in our experiments are summarized in Table 1. The network parameters are configured to mirror the bimodal latency characteristics often observed in congested O-RAN E2 interfaces [32], while the target mobility profiles cover a broad spectrum of geometric and stochastic motion patterns. For the TA-Gate mechanism, the monotonicity penalty coefficient λ is set to 10.0 to balance localization precision with physical-temporal consistency.

### 5.3. Jitter Modeling and Training Protocols

**Jitter Generation Logic:** The non-deterministic E2 backhaul jitter δ is modeled as an independent and identically distributed (i.i.d.) random variable for each sensing report, mimicking the per-packet queuing process in containerized Near-RT RIC environments. Specifically, the bimodal Gamma distribution follows $\delta ∼\pi \cdot \Gamma \left(k_{1},\theta_{1}\right)+\left(1-\pi \right)\cdot \Gamma \left(k_{2},\theta_{2}\right)$, with π=0.6. The first mode (10–50 ms) represents standard protocol processing, while the second mode (150–450 ms) simulates long-tail congestion (e.g., SCTP retransmissions). Both training and testing utilize the same sampling mechanism but are initialized with distinct random seeds to ensure that the model generalizes to stochastic temporal patterns rather than over-fitting a specific jitter sequence. We acknowledge that real cloud-native backhaul may exhibit temporal correlation, burstiness, and per-link heterogeneity beyond this i.i.d. model. However, the TA-Gate is designed to operate on the *realized* AoS value at each fusion snapshot rather than on the underlying delay distribution. Since the gate function w(τ) maps AoS to trust weight independently of how τ was generated, the framework is inherently agnostic to the delay process statistics—a property we consider advantageous for deployment across heterogeneous network conditions.

**Training methodology:** For each mobility model, we generated 2000 independent trajectory segments, totaling approximately $1.2\times 10^{5}$ sensing events. The data was partitioned into training, validation, and testing sets with a 7:1:2 ratio. During offline training, the framework utilizes global ground-truth (GT) coordinates to minimize the physics-constrained loss $L_{total}$. We employed the Adam optimizer ($lr=10^{-3}$) with a weight decay of $10^{-4}$ to prevent overfitting. An early stopping strategy with a patience of 100 epochs was applied based on the validation MSE. In the online inference phase, the model remains “time-blind” to the GT, relying solely on asynchronous telemetry $z_{i}$ and AoS metadata $\tau_{i}$ arriving at the Near-RT RIC.

All neural network components were implemented using the PyTorch Geometric library and executed on a standardized edge computing node (Intel i7-11800H CPU) to ensure that the reported latency measurements reflect a realistic RIC containerized runtime.

**RIC snapshot construction:** At each fusion instant $t_{k}$, the RIC constructs a single-snapshot graph by selecting, for each BS $b_{i}$, the most recent report whose arrival time satisfies $t_{arrival}^{\left(i\right)}\leq t_{k}$. This “latest-available” buffering policy requires no historical state backtracking and is consistent with the event-driven, stateless xApp execution model of the O-RAN Near-RT RIC. The AoS is then computed as $\tau_{i,k}=t_{k}-t_{obs}^{\left(i\right)}$, where $t_{obs}^{\left(i\right)}$ is the observation timestamp embedded in the E2 report header.

**Clock and timestamping assumptions:** We assume that all E2 Nodes and the Near-RT RIC share a common time reference (e.g., via GPS-disciplined clocks or IEEE 1588 PTP synchronization), such that $t_{obs}$ timestamps are globally consistent. This assumption is standard in O-RAN deployments where sub-microsecond synchronization is required for TDD coordination [1]. Under this assumption, the AoS $\tau_{i,k}$ faithfully reflects the true information age without clock-offset bias.

### 5.4. Baseline Methods

To ensure a fair and rigorous comparison, all evaluated algorithms operate on the identical raw telemetry stream $D_{i}=\left\{z_{i},\tau_{i},a_{i}\right\}$, where $z_{i}\in R^{2}$ is the coordinate estimate, $\tau_{i}$ is the AoS, and $a_{i}\in R^{2}$ represents the BS anchor coordinates. The baselines are implemented as follows:

- **Extended Kalman Filter (EKF) [7]:** A constant-velocity (CV) EKF is implemented as the primary recursive baseline, representing the practical configuration most likely deployed in real-time RIC xApps where computational overhead must be minimized.
- **EKF with OOSM [20]:** To evaluate the impact of Out-of-Sequence Measurement handling, we additionally implement an OOSM-equipped EKF that performs full state backtracking when delayed measurements arrive. This variant stores a rolling state history and re-propagates through all subsequent measurements upon receiving an out-of-order report, following the standard OOSM methodology [20].
- **AoS-weighted fusion:** A heuristic baseline that applies inverse-AoS weighting $w_{i}=1/\left(\tau_{i}+\epsilon \right)$ to compute a weighted centroid. It represents a straightforward deterministic linear-decay strategy to contrast with the learnable, non-linear logic of TA-Fusion.
- **Geometric WLS (G-WLS)** [6]: A non-linear solver that treats fusion as a spatial consensus problem. Notably, in our coordinate-level fusion setup, the WLS solution for minimizing squared Euclidean residuals reduces to a closed-form weighted centroid. Consequently, G-WLS provides an accuracy benchmark identical to AoS-weighted fusion, confirming that deterministic linear strategies reach a performance ceiling that cannot capture the complex spatio-temporal distortions inherent in O-RAN sensing.
- **Spatial GNN (time-blind):** Uses the same GATv2 backbone as TA-Fusion with a 3-dimensional node feature ${\left[z_{i}^{T},0\right]}^{T}\in R^{3}$, where the AoS channel is zeroed out. It ignores $\tau_{i}$ entirely to quantify the performance floor of purely spatial fusion.
- **AoS-aware GNN:** Represents the standard feature-level integration where AoS is appended as a passive input, resulting in a 3-dimensional vector ${\left[z_{i}^{T},{\tilde{\tau }}_{i}\right]}^{T}\in R^{3}$. This approach mirrors the temporal feature concatenation commonly adopted in spatial GNNs [21] and trajectory prediction models [23]. This baseline tests whether a GNN can inherently learn temporal decay without explicit physical guidance.
- **TA-Fusion (ours):** Extends the AoS-aware input with a duplicated AoS channel, resulting in a 4-dimensional vector ${\left[z_{i}^{T},{\tilde{\tau }}_{i},{\tilde{\tau }}_{i}\right]}^{T}\in R^{4}$. After graph attention aggregation, the TA-Gate computes physics-informed trust weights $w_{i}$ from the dedicated AoS channel and applies them as post-aggregation modulation (${\tilde{h}}_{i}=w_{i}\cdot h_{i}$) before global pooling. This active gating mechanism decouples reliable spatial representations from those degraded by temporal staleness.
- **Offline oracle (non-causal bound):** A non-causal WLS estimator that assumes zero network jitter (δ=0). By utilizing the instantaneous sensing coordinates $z_{i}\left(t_{obs}\right)$ directly, it defines the theoretical precision limit (3.28 m) imposed solely by physical measurement noise and geometric dilution of precision (GDOP).

**Fairness statement:** All GNN-based models share the identical GATv2 backbone (2 layers, 128 hidden units, and 4 heads in layer 1) and training budget (10,000 epochs). The variation in node feature dimensions (3-D to 4-D) stems from the inclusion of a dedicated AoS gate channel. TA-Fusion adds only the TA-Gate module (MLP_*gate*_: 1 → 32 → 1 with Tanh/Sigmoid activations, plus a learnable α scalar) on top of the AoS-aware baseline. As shown in Table 9, TA-Fusion maintains a comparable parameter footprint (≈45.2 K) to the AoS-aware GNN, ensuring that its 21.7% precision gain is attributable to the active gating logic rather than increased model capacity. Furthermore, the gate component ablation in Table 5 provides a controlled comparison against an unconstrained learned gate (MLP-only, without the physics prior or monotonicity penalty), directly isolating the contribution of each architectural element under identical training conditions.

**Remark on temporal GNN baselines:** We note that more expressive temporal architectures, such as temporal message-passing networks or transformer-based sequence models, represent potential alternatives. However, these models typically require sequential observation histories and incur significantly higher computational overhead ($O(T^{2}d)$ for self-attention over *T* time steps), making them less suitable for the single-snapshot, sub-10 ms inference regime targeted in this work. The TA-Gate is deliberately designed as a lightweight, per-snapshot mechanism that introduces minimal overhead (<0.2 ms) while achieving competitive temporal awareness.

### 5.5. Evaluation Metrics

The performance is quantified using the following metrics:

1. **Mean absolute error (MAE):** Reported as *Mean* in Table 2, it represents the average Euclidean distance between estimated and true positions.
2. **Root mean square error (RMSE):** Measures the overall localization precision. In this asynchronous fusion context, RMSE captures both the static spatial error and the asynchrony-induced trajectory warping.
3. **P90 error:** The 90th percentile of the error distribution, reflecting system reliability and the suppression of outlier errors under extreme jitter.
4. **Standard deviation (STD):** Quantifies the dispersion of the localization error, reflecting the stability and consistency of the estimator across all test samples.
5. **Trajectory roughness (rough.):** Quantifies the trajectory smoothness and temporal coherence described in Section 3.5 via the mean squared norm of the second-order trajectory difference:
  (12) $$R=\frac{1}{T-2}\sum_{t=2}^{T-1}{∥\left({\hat{p}}_{t+1}-{\hat{p}}_{t}\right)-\left({\hat{p}}_{t}-{\hat{p}}_{t-1}\right)∥}^{2}$$
  where ${\hat{p}}_{t}$ is the estimate at time *t*. While a lower R signifies a smoother path by suppressing non-physical jitter, it also reflects the fundamental trade-off between geometric consistency and the estimator’s tracking fidelity to high-dynamic maneuvers.
6. **Monte Carlo stability:** The mean RMSE evaluated across 100 randomized trials to ensure statistical significance and to evaluate the framework’s resilience against GDOP variability, with a dedicated analysis provided in Section 6.6.

## 6. Results and Discussion

### 6.1. Overall Localization Accuracy Analysis

The localization performance is evaluated under *Setting-1*, a standardized O-RAN sensing benchmark with a fixed BS geometry (N=8) and a bimodal E2 jitter profile (10–450 ms) that induces frequent out-of-order telemetry when $\delta >T_{s}$. Unless otherwise specified, all metrics reported in Table 2 are averaged over 100 independent Monte Carlo trials with different random seeds to ensure statistical significance.

As summarized in Table 2, TA-Fusion achieves an RMSE of 12.22 m, representing a substantial precision improvement over the AoS-aware GNN baseline (14.07 m). These results indicate that explicitly learning an AoS-to-trust mapping via the TA-Gate provides architectural benefits beyond treating AoS as a standard numerical input feature. For reference, the spatial GNN serves as a time-blind baseline to quantify the performance floor when Age-of-Sensing (AoS) metadata $\tau_{i}$ is entirely omitted, exhibiting an RMSE of 27.81 m.

**Limitations of traditional geometric fusion:** As shown in Table 2, traditional estimators such as EKF (128.53 m RMSE) and G-WLS (39.76 m RMSE) struggle to maintain tracking fidelity under asynchronous E2 jitter. The EKF suffers from irreversible state-lag as it lacks a mechanism to re-order or properly weight out-of-sequence telemetry within the required 10-ms-class execution budget. In particular, G-WLS yields results identical to AoS-weighted fusion (37.07 m MAE). This numerical equivalence arises because, in coordinate-level fusion, the WLS objective for minimizing squared Euclidean residuals mathematically reduces to a weighted centroid calculation. While stable, these linear baselines fundamentally fail to account for the non-linear spatio-temporal correlations induced by high-dynamic maneuvers, resulting in an MAE precision gap of over 300% compared to TA-Fusion (37.07 m vs. 9.24 m). The extreme P90 of AoS-weighted and G-WLS (506.89 m) reflects occasional geometric configurations where the weighted centroid degenerates under simultaneous high AoS variance across all nodes, producing catastrophic outliers that dominate the tail distribution.

**OOSM analysis:** To verify our design choice of omitting OOSM backtracking, we evaluate the EKF with full OOSM handling under the same benchmark conditions. The OOSM variant requires a mean inference latency of 28.64 ms (P95: 37.62 ms), exceeding the O-RAN sub-10 ms execution budget by nearly 3×. Moreover, the backtracking process actually degrades accuracy (RMSE 136.65 m vs. 128.53 m for the standard EKF), as re-propagation through heavily asynchronous measurements introduces compounding state estimation errors. In contrast, TA-Fusion achieves an RMSE of 12.22 m at 2.07 ms, confirming that the single-snapshot gating approach provides a fundamentally more suitable accuracy–latency trade-off for the O-RAN real-time regime.

**Precision distribution and SME mitigation:** Beyond the global RMSE gain, TA-Fusion delivers a more substantial 21.7% reduction in MAE (9.24 m vs. 11.80 m), highlighting superior precision in expected-case scenarios. This gain indicates that TA-Fusion effectively mitigates the stale-observation artifacts identified in Section 3.5. The 21.7% MAE reduction—contrasted with the more incremental 6.9% P90 improvement (20.01 m vs. 21.49 m)—indicates that the TA-Gate is most potent in nominal jitter regimes, where it can consistently prune the temporal aliasing that would otherwise bias the spatial estimate. The slightly higher STD (8.00 m) compared to the AoS-aware baseline reflects a deliberate bias-variance trade-off: by aggressively attenuating stale telemetry via the 0.4-safety margin, TA-Fusion reduces SME at the cost of the smoothing effect provided by temporal averaging. This prioritized tracking fidelity is essential for Near-RT RIC control loops requiring instantaneous state awareness.

**Trajectory integrity and warping mitigation:** A key highlight of the TA-Fusion framework is its superior trajectory consistency. As shown in Table 2, TA-Fusion achieves a roughness of 309.06 m^2^, a 22.3% reduction compared to the AoS-aware GNN (397.90 m^2^). While the passive AoS-aware baseline achieves relatively high precision, it suffers from significant trajectory “warping” or jitter, as it lacks a physical mechanism to prune stale observations that conflict with the instantaneous motion manifold. The active gating logic of the TA-Gate effectively suppresses these non-physical spatial jumps, ensuring that the estimated path remains smooth and continuous—a critical requirement for stable beamforming and link tracking in 6G ISAC systems.

**Accuracy–latency trade-off:** We additionally report an *Offline Oracle* as a non-causal theoretical upper bound (3.84 m RMSE) that assumes zero network jitter (δ=0). This Oracle represents the absolute physical limit imposed by measurement noise and geometry. In contrast, TA-Fusion maintains real-time inference (2.07 ms) while providing an optimal balance between precision and latency. This represents a 79.3% timing margin relative to the 10-ms O-RAN execution budget, suggesting compatibility with containerized xApp deployment under the tested conditions.

*Statistical significance:* To further validate the reliability of the performance gain, we conducted a paired *t*-test between TA-Fusion and the AoS-aware GNN across 100 Monte Carlo trials. The resulting *p*-value (p<0.01) indicates that the improvement is statistically significant. Furthermore, the 95% confidence interval (CI) for the RMSE of TA-Fusion is [12.10,12.34] m, confirming that the framework maintains consistent superiority under stochastic E2 jitter realizations.

### 6.2. Generalization Across Diverse Mobility Models

To evaluate the robustness of TA-Fusion across various target dynamics, we conduct evaluations using three distinct trajectory patterns: geometric (8-figure), transitional (zig-zag), and stochastic (random walk). The comparative results in Table 3 reveal a consistent performance hierarchy: spatial GNN → AoS-aware GNN → TA-Fusion.

Experimental results demonstrate that TA-Fusion maintains a stable performance advantage even in unseen mobility patterns. The reduction in both MAE and RMSE across all scenarios indicates that the framework improves average localization precision while suppressing large-magnitude errors induced by temporal aliasing. The roughness metric provides additional insight into trajectory integrity. In structured maneuvers like the 8-figure and zig-zag scenarios, TA-Fusion achieves significantly lower roughness compared to the AoS-aware baseline. This confirms that the TA-Gate effectively prunes the non-physical spatial “jumps” caused by stale telemetry during rapid transients, mitigating the “dragging effect” identified in our formalization. However, in the stochastic random walk scenario, TA-Fusion exhibits a slightly higher roughness (127.82 m^2^) than the baselines. This does not indicate instability; rather, it reflects superior tracking fidelity. In a random walk, the ground-truth trajectory is inherently non-smooth. While baselines achieve lower roughness through over-smoothed “lagging” (resulting in higher RMSE), TA-Fusion’s ability to maintain a lower RMSE (28.70 m) while following the stochastic shifts proves its agility and responsiveness to unpredictable dynamics.

Even in the random walk scenario, our framework yields an 8.0% MAE improvement over the AoS-aware GNN. This suggests that the TA-Gate captures a transferable decay prior of information utility rather than merely over-fitting to the curvatures of the training trajectory. The reduced error dispersion (STD) and P90 error across all unseen scenarios also reflect the structural robustness provided by our physics-informed gating mechanism, supporting reliable sensing in dynamic 6G environments.

### 6.3. CDF Analysis and Reliability

**Figure 6:**
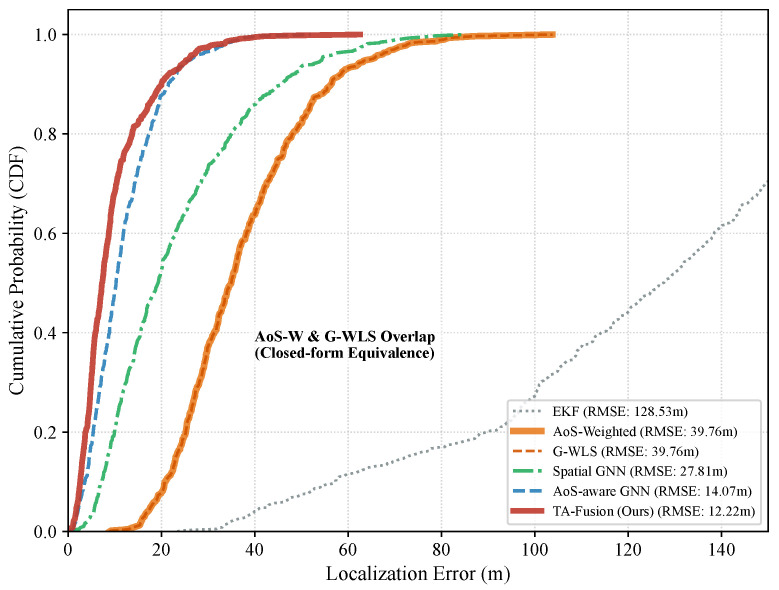
Cumulative distribution function (CDF) of localization errors under the Master Benchmark (Setting-1). The distributions are strictly consistent with the aggregate statistics reported in Table 2.

The cumulative distribution function (CDF) of localization errors is illustrated in Figure 6. This visualization provides a granular view of the per-sample errors recorded during the Master Benchmark evaluated in Section 6.1. By analyzing the error distribution across the entire test trajectory, we can evaluate the framework’s reliability beyond simple aggregate averages.

As observed, the TA-Fusion curve consistently maintains the leftmost position across the entire support, indicating a systemic improvement in localization reliability. Consistent with Table 2, the G-WLS and AoS-weighted curves exhibit a visual overlap; this further validates the closed-form mathematical equivalence between weighted least squares and weighted centroids discussed in Section 5.4.

At the critical P90 reliability threshold, TA-Fusion attains the tightest error bound of 20.01 m, effectively suppressing the “heavy-tail” outliers that plague both the Spatial and AoS-aware GNN baselines. Specifically, while the passive AoS-aware GNN relies on implicit temporal learning, the TA-Gate’s active trust modulation prevents stale telemetry from inducing trajectory “ghosting,” ensuring that the 90th percentile of errors remains strictly bounded. This “tail-pruning” effect is vital for maintaining sensing-control loop stability during peak jitter events, where unmanaged stale observations would otherwise trigger catastrophic handover oscillations or beam misalignment.

### 6.4. Ablation Study: The Impact of the TA-Gate

To isolate the contribution of the proposed TA-Gate, we conduct a controlled ablation study under an extreme mobility stress scenario (1.5/3.0 Hz maneuvering frequencies). This setup intentionally amplifies SME to evaluate the framework’s resilience under severe backhaul congestion. We analyze the three-stage evolution: from (i) time-blind spatial GNN to (ii) passive AoS-aware GNN, and finally to the full TA-Fusion model.

As summarized in Table 4, TA-Fusion delivers a decisive performance leap. Compared to the time-blind spatial GNN, our framework reduces the RMSE by 59.2%, effectively preventing the total system collapse observed when ignoring asynchronous SME. More importantly, introducing the TA-Gate mechanism provides a further 45.4% precision leap over the passive AoS-aware baseline. This two-stage jump confirms that while time-awareness is necessary, active physical gating is the primary catalyst for maintaining sub-35 m accuracy in highly volatile environments.

The roughness metrics reveal an important trade-off between temporal awareness and trajectory stability. While the time-blind spatial GNN appears smoother (1506.90 m^2^), this is a byproduct of its failure to track dynamic maneuvers (manifested as a massive 84.71 m RMSE). The passive AoS-aware GNN exhibits the highest roughness (2285.46 m^2^), confirming that simply adding AoS as a feature without physical constraints induces severe trajectory warping. TA-Fusion successfully breaks this trade-off, reducing the RMSE by 45.4% while simultaneously suppressing the roughness by 8.8% relative to the passive baseline, proving that the TA-Gate effectively filters out the non-physical “jitter” inherent in asynchronous sensing.

**TA-Gate component ablation:** To further dissect the contribution of each component within the TA-Gate, we evaluate three gating variants under the extreme stress scenario with independently trained models, as shown in Table 5. Note that the absolute RMSE values differ from Table 4 because each gate variant is trained from scratch with its own initialization; the comparison is therefore between gate configurations within this controlled experiment rather than across tables. The *Exp-only* gate uses only the exponential decay skeleton exp(−ατ) without the MLP calibration, while the *MLP-only* gate removes the physics prior and relies solely on the learned non-linear mapping.

The results reveal several key findings. First, the Exp-only gate performs *worse* than the passive baseline (RMSE 49.98 m vs. 42.33 m), indicating that a fixed exponential decay with a single learnable α is too rigid to capture the complex, non-stationary jitter dynamics of O-RAN backhaul. Second, the MLP-only gate achieves strong performance (RMSE 34.68 m), demonstrating the importance of non-linear adaptability. Third, the full hybrid TA-Gate achieves the best overall results (RMSE 34.26 m, roughness 1447.68 m^2^), confirming that the exponential skeleton contributes primarily as a *structural regularizer*: while the MLP provides the adaptive capacity, the physics prior constrains the learned mapping to respect the monotonic decay of information utility, yielding the lowest trajectory roughness among all variants.

**Role of the monotonic prior:** The enforcement of $L_{mono}$ ensures that trust assignments remain physically consistent across all jitter regimes. While unconstrained models may occasionally over-trust stale data due to spurious spatial correlations, TA-Fusion’s hybrid structure maintains both interpretability and structural robustness. This is particularly evident during sharp maneuvers, where the model effectively rejects spurious estimates pulled away by stale but geometrically plausible reports. By anchoring the fusion process to the monotonic decay of information utility, TA-Fusion ensures that the sensing results remain physically grounded, directly contributing to the reduced trajectory roughness and operational reliability required for high-speed motion orchestration in 6G networks.

### 6.5. Sensitivity Analysis of Safety Margin β

To justify the selection of the safety margin β, we conduct a sensitivity analysis under an extreme mobility stress test with independently trained models for each β value. This parameter defines the structural lower bound of information trust ($w_{min}=\beta$), ensuring that even stale telemetry nodes provide a baseline contribution to the geometric constraints. The results are summarized in Table 6. Note that the β=0.4 entry (RMSE 34.45 m) differs slightly from the full TA-Gate result in Table 5 (RMSE 34.26 m) due to independent training runs with different random initialization.

The empirical results reveal a pronounced U-shaped error profile with respect to β, confirming that this parameter critically governs the accuracy–robustness trade-off. When β=0, the gating mechanism can completely suppress nodes with high AoS, eliminating their spatial contribution entirely. This causes a catastrophic RMSE of 204.26 m as the fusion center loses critical geometric anchors, suffering from severe GDOP. Even a moderate floor of β=0.2 reduces the RMSE to 96.76 m, demonstrating that partial anchor preservation already provides substantial geometric recovery.

The optimal performance at β=0.4 (RMSE 34.45 m) confirms that retaining 40% baseline trust is the ideal operating point for this high-dynamic environment. It preserves the geometric stability of the sensing graph while aggressively filtering the most severe temporal staleness. Conversely, increasing β to 0.6 or 0.8 leads to progressively worse performance (RMSE 131.08 m and 239.46 m, respectively), as stale observations retain excessive influence and introduce severe ghosting artifacts. The strong asymmetry of the U-curve—where both extremes degrade by an order of magnitude—underscores the sensitivity of asynchronous fusion to this structural hyperparameter and validates the chosen operating point of β=0.4.

### 6.6. Statistical Stability: Monte Carlo Simulations

**Figure 7:**
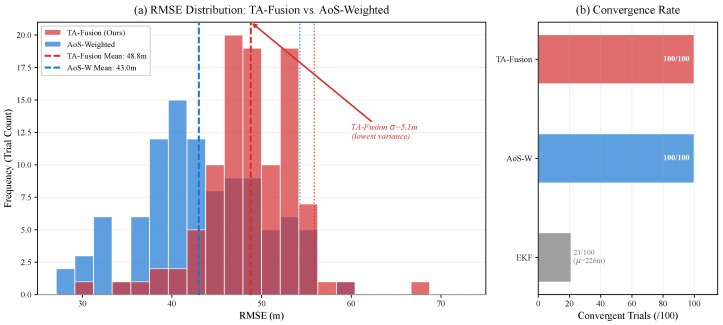
Monte Carlo robustness evaluation across 100 randomized BS layouts. (**a**) RMSE distributions of TA-Fusion and AoS-weighted centroid, showing comparable mean errors but lower variance for TA-Fusion (σ=5.08 m vs. 6.87 m). (**b**) Convergence rates: TA-Fusion and AoS-weighted achieve 100% convergence, while EKF diverges in 79% of trials (mean RMSE =226 m).

The statistical distribution of the localization error across 100 Monte Carlo trials with randomized geometries is illustrated in Figure 7. To contextualize TA-Fusion’s robustness, we compare against the AoS-weighted centroid and EKF baselines under the same randomized layouts, as summarized in Table 7.

Several findings merit discussion. First, the absolute RMSE increases from 12.22 m (Table 2) to ∼49 m, which is primarily governed by the GDOP inherent in unoptimized, random BS layouts, rather than algorithmic failure. Second, the EKF catastrophically diverges in 79% of trials (convergence rate 21/100), confirming that recursive filters without OOSM handling are fundamentally incompatible with the combination of asynchronous sensing and unknown geometries. Third, while the AoS-weighted centroid achieves a lower mean RMSE (43.00 m) than TA-Fusion (48.80 m) in this randomized setting—an expected result since analytical methods require no training and naturally adapt to any geometry—TA-Fusion exhibits the *lowest standard deviation* (5.08 m vs. 6.87 m), indicating the most consistent and predictable performance across diverse topologies. This reduced variance is a critical operational property: in deployment scenarios where the BS geometry is fixed and known (as in the benchmark setting of Table 2), TA-Fusion retains its substantial precision advantage (12.22 m vs. 39.76 m), while in unseen geometries it gracefully degrades to an error level comparable to—and more stable than—the analytical baseline.

### 6.7. Robustness Analysis Against Information Staleness

**Figure 8:**
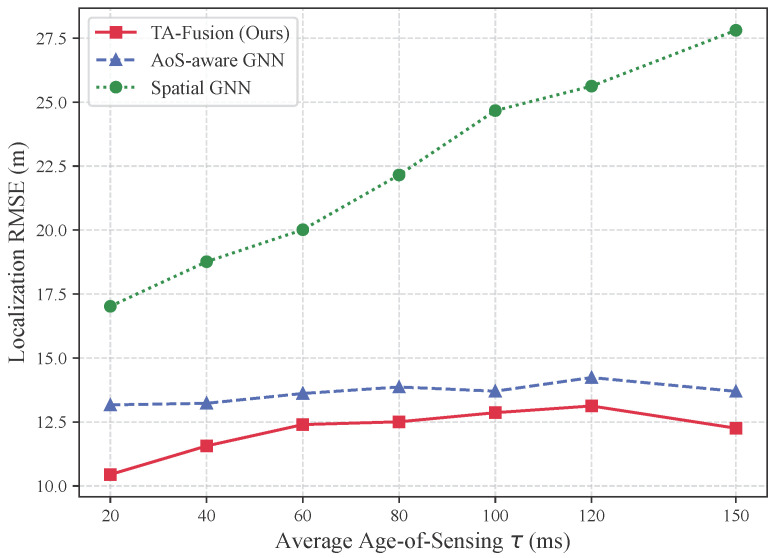
Localization RMSE versus average Age-of-Sensing (AoS). The comparison between TA-Fusion and AoS-aware GNN illustrates improved robustness and bounded degradation under increasing temporal staleness.

To evaluate sensitivity to escalating temporal staleness, we sweep the average AoS from 20 ms to 150 ms while maintaining identical sensing graph size and noise statistics. Unlike the bimodal jitter setting in Table 2, this experiment isolates the impact of mean delay. Figure 8 depicts the resulting RMSE trends.

As the average AoS increases sevenfold (20 → 150 ms), the RMSE of TA-Fusion increases only from 11.20 m to 13.14 m, corresponding to a net rise of 1.94 m. Even at the observed error peak (60 ms), the error remains bounded at 13.42 m. Minor non-monotonic fluctuations (e.g., the peak around 60 ms) are expected due to finite-sample variability and the interaction between stochastic jitter realizations and trajectory maneuvers; nevertheless, the overall curve remains tightly bounded, demonstrating a practical robustness plateau. This limited sensitivity indicates that the proposed TA-Gate effectively performs selective trust reweighting: as global delay grows, relatively fresher observations are emphasized to anchor the localization estimate.

In contrast, the AoS-aware GNN exhibits higher error and larger fluctuations across the same AoS regime. These results demonstrate that explicitly modeling the monotonic decay of information trust improves robustness under sustained backhaul congestion, ensuring stable spatial consistency even as average delay escalates.

### 6.8. Scalability Analysis Across Network Sizes

To evaluate the framework’s behavior under varying network densities, we conduct a scalability study across N∈{4,6,8,12} base stations—covering the typical range of O-RAN macro-cell cluster configurations where a Near-RT RIC serves 4–12 gNB sectors [1]. For each *N*, we evaluate 10 randomized BS layouts with *k*-NN connectivity k=min(4,N−1). Each configuration is independently trained and evaluated under the standard bimodal jitter model. Table 8 summarizes the results.

Several findings merit discussion. First, TA-Fusion consistently outperforms the AoS-weighted baseline across all tested network scales, with mean gains ranging from 25.2% to 48.2%. Second, the AoS-weighted RMSE decreases monotonically with *N* (39.98 → 35.58 m), reflecting the expected GDOP improvement from denser geometries—the mean GDOP decreases from 1.13 at N=4 to 0.62 at N=12, confirming that additional base stations improve the geometric conditioning of the fusion problem. Third, the higher standard deviation of TA-Fusion compared to the analytical baseline reflects the learned model’s sensitivity to specific BS geometries—consistent with the Monte Carlo findings in Table 7. In deployment, where the BS layout is fixed and known, this variance vanishes as the model is trained on the actual geometry.

The non-monotonic variation in TA-Fusion’s RMSE across *N* (e.g., N=8 exhibiting higher RMSE than N=6) contrasts with the monotonic trend observed for the analytical baseline, suggesting that the variation is driven by training stochasticity rather than geometry. Specifically, N=8 exhibits a high standard deviation (12.26 m) due to individual outlier runs with unfavorable initialization, whereas N=6 achieves the lowest standard deviation (5.02 m) across its 10 trials. A correlation analysis across all 40 runs confirms this: the correlation between GDOP and TA-Fusion RMSE is weak (ρ=0.27), indicating that geometry explains only a small fraction of the variance. The key finding is that TA-Fusion maintains a consistent precision advantage over the analytical baseline at every tested scale within the practical O-RAN deployment range.

### 6.9. Computational Complexity and Real-Time Profiling

To verify the real-time feasibility of TA-Fusion within the Near-RT RIC environment, we conducted end-to-end profiling on an edge-grade CPU (Intel i7-11800H). As summarized in Table 9, the inference latency is 2.07 ms, with the 95th percentile (P95) strictly bounded at 2.35 ms.

To contextualize these metrics, we compare TA-Fusion against both the baseline spatial GNN and the AoS-aware GNN paradigm. The results demonstrate that while the transition to an AoS-aware architecture yields the most significant precision leap, the inclusion of our physics-informed TA-Gate introduces a marginal parameter overhead of only 3.2% relative to the base AoS-aware model.

This lean computational profile provides a substantial timing margin of 79.3% relative to the 10-ms Near-RT RIC execution budget [33]. Such a buffer is critical for accommodating OS-level scheduling jitter or containerized resource contention in cloud-native O-RAN deployments. Furthermore, the model maintains a compact runtime memory footprint of approximately 12.4 MB. Given the sparse sensing graph structure (|E|≈32), the computational complexity scales linearly with the number of nodes. This confirms that the TA-Fusion framework is fully compatible with the sub-10 ms execution envelope required for delay-sensitive ISAC xApps, such as predictive beam-tracking and rapid handover orchestration.

### 6.10. Interpretation of Localization Accuracy

The achieved localization accuracy should be interpreted in the context of network-native sensing objectives and application requirements. Unlike automotive radar systems designed for centimeter-level object localization, infrastructure-based ISAC sensing primarily aims to provide network-scale spatial awareness across large coverage areas. In such scenarios, meter-level localization accuracy is generally sufficient for infrastructure-level sensing applications, including mobility management, proactive handover, traffic monitoring, and resource orchestration. In many network-side applications, the decision granularity typically spans tens to hundreds of meters, which makes meter-level localization accuracy practically sufficient for infrastructure-level sensing tasks.

Recent studies on cooperative ISAC sensing architectures have similarly emphasized infrastructure-level situational awareness rather than high-precision radar localization [2,3]. Furthermore, theoretical analyses of cooperative sensing networks indicate that achievable localization accuracy is fundamentally constrained by sensing geometry, synchronization conditions, and communication waveform limitations [19]. Therefore, the primary contribution of this work lies not in pursuing centimeter-level localization precision, but in establishing a delay-resilient sensing fusion paradigm capable of maintaining stable spatial inference under severe network-layer asynchrony.

## 7. Conclusions

This paper addresses the fundamental challenge of temporal asynchrony in ISAC-enabled O-RAN systems. We presented the TA-Fusion framework, which treats Age-of-Sensing as an active post-aggregation gating signal within a graph attention architecture, providing a resilient fusion mechanism for mitigating spatial artifacts induced by non-deterministic backhaul jitter. By integrating a physics-informed TA-Gate mechanism with a graph attention backbone, the proposed TA-Fusion framework structurally formalizes the coupling between network-layer information freshness and physical-layer spatial consistency. The experimental evaluation demonstrates that this architectural shift provides substantial precision enhancements over conventional spatial GNNs, while the TA-Gate further ensures robust tracking fidelity in extreme jitter scenarios where traditional iterative solvers reach a performance floor due to their static weighting logic. The framework’s computational efficiency ensures high compatibility with the stringent sub-10 ms execution targets required for delay-critical O-RAN sensing operations. Furthermore, the analysis confirms that TA-Fusion offers a stable and bounded error distribution across randomized deployment geometries without the need for manual recalibration. These results suggest that the proposed framework is a robust and scalable sensing component suitable for stochastic, containerized xApp environments in 6G networks. It is important to note that the objective of network-native sensing differs fundamentally from high-resolution radar localization systems designed for autonomous driving. Instead, infrastructure-based ISAC sensing primarily aims to provide network-scale situational awareness across large coverage areas, where meter-level spatial accuracy is generally sufficient for higher-layer network orchestration tasks [2,3].

Despite these improvements, certain limitations remain. This study assumes resolved data association and primarily focuses on single-target scenarios; extending the framework to handle multi-target tracking in cluttered sensing environments is a high priority. While the scalability analysis (Table 8) confirms consistent performance gains across the practical O-RAN range of N=4 to 12, ultra-dense deployments (N≥32) remain an open question. As analyzed in Section 4.4, the inference complexity scales as O(L·Nkd) for sparse *k*-NN graphs, suggesting that moderate scaling is feasible; however, ultra-dense deployments may require dynamic graph sampling or hierarchical aggregation to maintain real-time constraints. Additionally, the evaluation relies entirely on synthetic simulations. While the bimodal Gamma jitter model captures the key statistical characteristics of O-RAN E2 latency, real deployments may exhibit correlated, bursty, or link-heterogeneous delay patterns that require further validation on hardware-in-the-loop testbeds.

Future research will incorporate *Uncertainty Quantification (UQ)* to provide formal confidence intervals for state estimates, an essential feature for safety-critical V2X applications. We also intend to explore cooperative sensing with mobile E2 Nodes, such as UAV-mounted base stations, and validate the xApp performance on a hardware-in-the-loop (HiL) testbed to further align simulation results with real-world O-RAN operating conditions.

## Figures and Tables

**Table 1:** Simulation and model hyper-parameters.

Parameter	Value
*Sensing Environment*	
Sensing area size	1000×1000 m
Number of base stations (*N*)	8
Number of neighbors ($k_{NN}$)	4
Sensing frequency ($f_{s}$)	50 Hz
Reporting interval ($T_{s}$)	20 ms
Measurement noise (σ)	1.0 m
Aggregate E2 latency (δ)	10–450 ms (truncated)
Latency mixture parameters	$\pi =0.6,\;k_{1}=2,\;\theta_{1}=15,\;k_{2}=15,\;\theta_{2}=20$
Dataset size (total events)	≈$1.2\times 10^{5}$
*Model and Training*	
GNN hidden dimensions	128 (2 layers)
Optimizer	Adam ($\beta_{1}=0.9,\;\beta_{2}=0.999$)
Learning rate/weight decay	$10^{-3}$/$10^{-4}$
Training batch size	128 (graph snapshots)
Inference batch size	1 (sequential streaming)
Training epochs/patience	10,000/100
AoS normalization factor (γ)	10.0
Monotonicity sampling range ($\tilde{\tau }$)	[0,4.5]
Monotonicity penalty coeff. (λ)	10.0
AoS decay constant (α)	2.0 (initial)
Node feature dim. (spatial/AoS-aware/TA-Fusion)	3-D/3-D/4-D

**Table 2:** Master performance comparison: localization precision and real-time profiling under E2 jitter. All spatial error metrics are reported in meters (m).

Algorithm	MAE (m)	RMSE (m)	P90 (m)	STD (m)	Rough. ($m^{2}$)	Latency	Real-Time?
EKF [7]	121.61	128.53	169.59	41.62	83.81	0.24 ms	Yes
AoS-weighted ^†^	37.07	39.76	506.89	56.36	14.39	0.97 ms	Yes
G-WLS [6] ^†^	37.07	39.76	506.89	56.36	14.39	0.95 ms	Yes
Spatial GNN [8]	23.06	27.81	45.81	15.55	467.96	1.87 ms	Yes
AoS-aware GNN	11.80	14.07	21.49	7.67	397.90	1.89 ms	Yes
**TA-Fusion (Ours)** ^‡^	**9.24**	**12.22**	**20.01**	8.00	**309.06**	2.07 ms	Yes
*Offline Oracle*	*3.28*	*3.84*	*6.06*	*2.00*	*107.77*	*>500 ms*	*No*

^†^ In coordinate-level fusion, the AoS-weighted WLS solution is mathematically equivalent to the weighted centroid, resulting in identical performance. ^‡^ Represents a 21.7% MAE improvement and a 13.1% RMSE improvement relative to the AoS-aware GNN baseline.

**Table 3:** Generalization performance comparison across diverse mobility scenarios.

Scenario	Model	MAE (m)	RMSE (m)	P90 (m)	Rough. ($m^{2}$)	Gain (MAE, %)
8-figure (Trained)	Spatial GNN	23.06	27.81	45.81	467.96	—
	AoS-aware GNN	11.80	14.07	21.49	397.90	—
	**TA-Fusion (Ours)**	9.24	12.22	20.01	309.06	21.7%
Zig-zag (Unseen)	Spatial GNN	37.98	43.87	71.49	56.13	—
	AoS-aware GNN	35.37	39.41	61.62	119.18	—
	**TA-Fusion (Ours)**	31.55	34.98	54.97	114.74	10.8%
Random (Unseen)	Spatial GNN	33.13	36.49	55.15	91.75	—
	AoS-aware GNN	28.32	31.15	45.84	103.92	—
	**TA-Fusion (Ours)**	26.06	28.70	43.00	127.82	8.0%

**Table 4:** Ablation results under extreme stress: from spatial fusion to TA-Fusion.

Model Configuration	MAE (m)	RMSE (m)	Rough. ($m^{2}$)	Gain (%)
Spatial GNN	75.30	84.71	1506.90	—
AoS-aware GNN	54.39	63.24	2285.46	25.3% *
**TA-Fusion (Ours)**	28.01	34.54	2083.68	45.4%

* Gain of AoS-aware over spatial GNN (RMSE). The 45.4% RMSE gain of TA-Fusion over the AoS-aware baseline represents the additional precision contributed by the proposed TA-Gate mechanism.

**Table 5:** TA-Gate component ablation under extreme stress.

Gate Configuration	MAE (m)	RMSE (m)	Rough. ($m^{2}$)
AoS-aware GNN (no gate)	36.14	42.33	1458.98
Exp-only gate	43.60	49.98	1808.38
MLP-only gate	29.32	34.68	1498.28
**Full TA-Gate (Ours)**	**28.40**	**34.26**	**1447.68**

**Table 6:** Sensitivity analysis of the safety margin β on localization accuracy.

Safety Margin β	MAE (m)	RMSE (m)	Observation
0.0	193.48	204.26	Over-suppression (Anchor Loss)
0.2	90.55	96.76	Moderate suppression
**0.4**	**28.06**	**34.45**	**Optimal Balance**
0.6	122.98	131.08	Under-suppression (Ghosting)
0.8	226.68	239.46	Excessive floor (Severe Ghosting)

**Table 7:** Monte Carlo comparison across 100 randomized BS layouts.

Method	Mean RMSE (m)	Std (m)	P95 (m)	Convergence
EKF	225.61	27.75	269.58	21/100
AoS-weighted	43.00	6.87	54.24	100/100
**TA-Fusion (Ours)**	**48.80**	**5.08**	**55.87**	**100/100**

Convergence: trials with RMSE < 200 m. All models trained on fixed benchmark layout and evaluated on unseen randomized geometries.

**Table 8:** Scalability analysis: localization RMSE across network sizes (10 randomized layouts per *N*).

*N*	*k*	TA-Fusion RMSE (m)	AoS-W RMSE (m)	Gain (%)	GDOP
4	3	29.89±13.54	39.98±2.01	25.2	1.13
6	4	19.78±5.02	38.20±2.01	48.2	0.92
8	4	25.41±12.26	37.22±1.13	31.7	0.75
12	4	24.84±5.71	35.58±0.86	30.2	0.62

All values are mean ± std over 10 independent randomized BS layouts. GDOP is the mean value at the area center. The benchmark result in Table 2 (RMSE = 12.22 m at N=8) uses a fixed optimized layout; the higher absolute values here reflect the GDOP penalty of random geometries.

**Table 9:** Comparative profiling: resource utilization and efficiency.

Metric	Spatial GNN	AoS-Aware GNN	TA-Fusion (Ours)
Avg. Latency (ms)	1.87	1.89	2.07
Trainable Params	42.1 K	43.8 K	45.2 K
Storage Size (KB)	172	178	185
RMSE (m)	27.81	14.07	**12.22**

## Data Availability

The simulation code and data supporting the findings of this study are available from the corresponding author upon reasonable request.
